# Gabapentin for phantom limb pain after amputation in pediatric oncology: a systematic review protocol

**DOI:** 10.1186/s13643-020-01571-8

**Published:** 2021-01-13

**Authors:** Shuang Jiang, Meng-meng Zhou, Rong Xia, Jing-hui Bai, Li-hui Yan

**Affiliations:** 1 Department of Internal Medicine (Psychologic Clinic), Cancer Hospital of China Medical University, Shenyang, 110042 China; 2 Department of Psychology Clinic, Shengjing Hospital of China Medical University, Shenyang, 110022 China; 3 Department of Internal Medicine (VIP Ward), Cancer Hospital of China Medical University, Shenyang, 110042 China; 4 Department of Internal Medicine (Pain Clinic), Cancer Hospital of China Medical University, No. 44 Xiaoheyan Road, Dadong District, Shenyang, 110042 China

**Keywords:** Phantom limb pain, Amputation, Gabapentin, Efficacy, Safety

## Abstract

**Background:**

Phantom limb pain (PLP) is a prevalent problem for children after amputation because of the chemotherapy treatment. Gabapentin is a potential option to manage PLP after amputation in pediatric oncology. However, no systematic review specifically investigated this topic. Thus, this study aims to appraise the efficacy and safety of gabapentin for post-amputation PLP in pediatric oncology.

**Methods:**

Electronic databases (Cochrane Library, MEDLINE, EMBASE, Web of Science, CINAHL, PsychINFO, Scopus, WANGFANG, and Chinese Biomedical Literature Database) will be systematically searched from the beginning to the present without limitations to publication status and language. Primary outcome is pain intensity. Secondary outcomes are analgesic drug consumption, sleep quality, depression, anxiety, health-related quality of life, and adverse events. The treatment effect of all dichotomous outcome data will be estimated as risk ratio and 95% confidence intervals (CIs) and that of continuous outcome data will be calculated as mean difference or standardized mean difference and 95% CIs. Methodological quality of randomized controlled trials (RCTs) will be assessed using Cochrane risk of bias tool and that of case-controlled studies (CCSs) will be appraised using Newcastle-Ottawa Tool. Statistical analysis will be conducted using RevMan 5.3 software.

**Discussion:**

This study will summarize up-to-date high-quality RCTs and CCSs to assess the efficacy and safety of gabapentin for PLP after amputation in pediatric oncology. The findings of this study will help to determine whether or not gabapentin is effective and safe for children with PLP after amputation.

**Systematic review registration:**

INPLASY202060090

**Supplementary Information:**

The online version contains supplementary material available at 10.1186/s13643-020-01571-8.

## Background

Phantom limb pain (PLP) is clinically defined as the perception of pain or discomfort in a missing or amputated limb [1, 2]. Its symptoms vary from sharp to tingling [3, 4]. PLP in children usually occurs few days in the afternoon or evening, daily, or weekly after limb amputation and typically lasts from seconds to minutes [5, 6]. In pediatric population, the most common causes of PLP are vascular etiologies, trauma, cancer/malignancy, and congenital conditions [6]. It has been estimated that its prevalence rate ranges between 12 and 83%, based on different study reports [7–12]. In the pediatric oncology population, its prevalence rate varies from 48 to 90% [7, 10, 13, 14]. Previous studies have shown that PLP can lead to many problems, including depression, anxiety, and stress, which significantly decreases the health-related quality of life in such patients [9, 15–17]. Currently, its pathophysiology is complex and its mechanisms are still poorly understood.

For pediatric oncology patients, amputation is the most commonly utilized management for tumor control in osteosarcoma bone cancer [18, 19]. It has been reported that the administration of chemotherapy before amputation surgery is one of the most risk factors to develop PLP [7]. Currently, no curative treatments for PLP are available, and its therapy mainly focuses on symptomatic control [7, 20]. The most common drug for PLP is morphine [21]. However, it can only relieve PLP in about 50% of patients, and there are still about 30% of cases having poor response to morphine [22]. Thus, alternative therapy is urgently needed.

Gabapentin, also known as an anticonvulsant or anti-epileptic drug, is a structural analog of gamma-aminobutyric acid (GABA) agonist, which is utilized for the treatment of epilepsy [23, 24]. It is also used for a variety of neuropathic pain conditions management with an analgesic effect [25]. Studies have suggested that gabapentin has been shown to be effective in several certain types of pain, such as PLP [26–28]. However, its mechanism of action has not been fully explored. Proposed mechanism on the action of gabapentin may enhance GABA release, which can employ an inhibitory effect on pain neurotransmission [29]. Several studies reported that gabapentin can effectively treat PLP after amputation in pediatric oncology [30–33]. However, there is no systematic review that specifically addresses this topic. Thus, this systematic review seeks to provide a comprehensive and systematic review of the best current evidence regarding gabapentin for PLP after amputation in pediatric oncology.

### Objective

The aim of this review is to systematically appraise the evidence of gabapentin for PLP after amputation in pediatric oncology.

This study sought to answer the following questions:

1. Does gabapentin relieve PLP after amputation in pediatric oncology?
2. Is gabapentin safe for the treatment of PLP after amputation in pediatric oncology?

## Methods and analysis

This study has been registered on International Platform of Registered Systematic Review and Meta-analysis Protocols with registration number of INPLASY202060090 (https://www.doi.org;DOI:10.37766/inplasy2020.6.0090). It is designed according to the Guidelines of Preferred Reporting Items for Systematic Reviews and Meta-Analysis (PRISMA) Protocol Statement (Additional file 1) [34, 35]. Any modifications will be reported in the protocol during the systematic review performance.

### Search strategy

We will systematically retrieve electronic databases (Cochrane Library, MEDLINE, EMBASE, Web of Science, CINAHL, PsychINFO, Scopus, WANGFANG, and Chinese Biomedical Literature Database) from the inception to the present without restrictions to publication status and language. The search strategy is built using keywords including “neoplasms,” “pain,” “cancer pain,” “phantom limb,” “pain intensity,” “neuropathic,” “chronic pain,” “gabapentin,” “anti-epileptic drug,” “anticonvulsant,” “neurontin,” “gralise,” “GABA analogs,” “random,” “allocation,” “placebo,” “sham,” “blind,” “control trial,” “case-control,” “case control,” “case-comparison,” “case-referent,” “clinical trial,” “observational study,” “study,” and “trial.” A detailed search strategy for Cochrane Library is presented in Table 1. Similar search strategies for other electronic databases will be adapted and applied. Translations will be performed when necessary in Chinese databases. At the same time, we will examine unpublished and ongoing work in clinical trial registry, conference proceedings, and reference lists of eligible studies. Two independent reviewers (X-R and B-JH) will carry out the whole process of systematic searches. Any disagreements will be solved by discussion with the help of a third reviewer (Y-LH). A consistent decision will be reached after discussion.

**Table 1:** Search strategy of Cochrane Library database

Number	Search terms
1	MeSH descriptor: (neoplasms) explode all trees
2	MeSH descriptor: (pain) explode all trees
3	MeSH descriptor: (cancer pain) explode all trees
4	MeSH descriptor: (phantom limb) explode all trees
5	((neoplasms*) or (cancer*) or (tumor*) or (phantom*) or (limb*) or (pain*) or (pain intensity*) or (neuropathic*) or (cancer pain*) or (chronic pain*)):ti, ab, kw
6	Or 1-5
7	MeSH descriptor: (gabapentin) explode all trees
8	((gabapentin*) or (anti-epileptic drug*) or (anticonvulsant*) or (neurontin*) or (gralise*) or (GABA analogs*)):ti, ab, kw
9	Or 7-8
10	MeSH descriptor: (randomized controlled trials) explode all trees
11	MeSH descriptor: (case-control studies) explode all trees
12	((random*) or (allocation*) or (placebo*) or (sham*) or (blind*) or (control trial*) or (case-control*) or (case control*) or (case-comparison*) or (clinical trial*) or (observational study*) or (study*) or (trial*)):ti, ab, kw
13	Or 10-12
14	6 and 9 and 13

### Eligibility criteria for study selection

#### Types of studies

Primary studies including randomized controlled trials (RCTs) and case-controlled studies (CCSs) will be included. Eligible studies are those that are disseminated up to the present in any language and publication status and those that report one of the outcomes of interest. However, we will exclude animal study, review, editorial letter, case report, case series, non-clinical trial, and uncontrolled study.

#### Types of participants

We will include studies involving pediatric oncology patients (under 18 years old) with confirmed bone cancers, irrespective of race, sex, and duration of PLP. In addition, eligible participants are those who suffered from PLP after amputation in pediatric oncology. However, we will excluded participants with multiple metastases, abnormal renal and hepatic function, and allergy to gabapentin and study drugs. In addition, we will also not consider patients with pain caused by other diseases, except PLP.

#### Types of interventions

We will consider studies for inclusion that report outcomes in pediatric oncology patients using any forms of gabapentin.

#### Types of comparators

There will be no limitations to the comparators, such as routine medication, and placebo. However, we will exclude any comparators that involved any types of gabapentin.

#### Types of outcomes

##### Primary outcome

Pain intensity (any pain scale reported in the trial, such as visual analogue scale)

##### Secondary outcome

- Analgesic drug consumption (any analgesic medication reported in the trial)
- Sleep quality (any related scale reported in the trial, such as Medical Outcomes Study Sleep Scale)
- Depression (any associated score reported in the trial, such as Zung Depression Scale)
- Anxiety (any relevant tool reported in the trial, such as Beck Anxiety Inventory)
- Health-related quality of life (any relevant tool reported in the trial, such as 36-Item Short Form Survey)
- Adverse events (any records reported in the trial)

### Study selection

Records retrieved from the literature sources will be imported to a reference management, and duplicates will be eliminated. Two independent authors (J-S and Z-MM) will carry out a broad scan of study titles and abstracts; and unrelated studies will be removed. Then, full-paper of potential eligible studies will be checked to make sure whether they fulfill all eligibility criteria. Any divergences between two authors will be solved by consulting a third author (X-R), and a final decision will be reached after the discussion. Reasons for all excluded studies at different stages will be recorded. The results of study selection process will be summarized in a PRISMA flowchart.

### Data extraction and management

Data from selected RCTs and CCSs will be transferred from their original presentation to a standard form with each included study receiving a reference code. If necessary, we will also extract indirect data from figures and charts.

For all included RCTs and CCSs, two authors (J-S and Z-MM) will independently obtain the data from eligible trials according to the predefined data extraction sheet developed specifically for this study. Any opposite views regarding the data extraction will be resolved by discussion with the help of another author (X-R), and we will make a final consistent decision. The extracted information consists of study characteristics (such as country, title, language, publication time, and funding source), patient characteristics (such as age, gender, and diagnostic criteria), study design (such as randomization details, blind, and lost to follow-up), intervention and control details (such as treatment types, duration, and number and length of sessions), and outcomes, safety, and other related information (such as confounding factors).

### Study quality assessment

The study quality of each eligible study will be examined by two independent authors (X-R and B-JH) using Cochrane Collaboration’s Risk of Bias Tool for RCTs [36] and Newcastle-Ottawa Scale for CCSs [37], with predetermined criteria. RCTs will be assessed on seven aspects, and each one is further rated as high, unclear, or low risk of bias [36]. CCSs will be appraised on three broad perspectives with eight specific items [37]. Any doubt between two authors will be answered with the help of a third author (Y-LH) through discussion, and a final consistent decision will be made.

### Dealing with missing data

We will contact corresponding authors of primary studies to obtain any missing or insufficient or unclear data by email or fax. If we can not achieve those data, we will analyze available data using Intention-To-Treat approach and will discuss its potential affects in the manuscript.

### Assessment of reporting bias

If there is a minimum of 10 trials in any meta-analysis, we will examine reporting bias using a funnel plot [38], and symmetry of the funnel plot will be performed using Egger’s regression test [39].

### Data synthesis

We will use RevMan 5.3 software to synthesize and analyze all outcome data. We plan to carry out separate analysis based on types of study, including RCTs and CCSs. We will calculate the treatment effect of dichotomous data using risk ratio and 95% confidence intervals (CIs) and that of continuous data using mean difference (MD) or standardized MD and 95% CIs. We will examine heterogeneity using *I*^2^ statistic, and we will undertake statistical pooling on groups of trials which are considered to be sufficiently similar [40, 41]. Where heterogeneity is low or minor (*I*^2^ ≤ 25%), we will utilize a fixed-effect model to pool the data; if heterogeneity is moderate (25% < *I*^2^ ≤ 75%), we will apply a random-effect model to synthesize the data, and if heterogeneity is obvious (*I*^2^ > 75%), we will not pool the data [40]. Meta-analysis will be carried out based on the sufficient homogeneity regarding on participant characteristics, types of intervention and outcome, and comparability between methods and ability to aggregate data. A narrative synthesis of eligible trials will be performed if the extracted data is too diverse to fulfill the threshold for meta-analytic approach. We will build a “summary of findings” table for the outcomes, and we will appraise evidence quality of primary outcome using Grading of Recommendations Assessment, Development and Evaluation [42, 43], which covers five aspects of risk of bias, imprecision, consistency of effect, indirectness, and publication bias.

### Subgroup analysis

We will carry out subgroup analysis to test the sources of significant heterogeneity based on the following:

- Studies at low risk of bias compared to high risk of bias
- Studies stratified according to different forms of gabapentin, such as single modality and combined managements
- Studies stratified based on the control treatments
- Studies stratified in accordance with the different geographical regions, and outcomes at different time points

### Sensitivity analysis

We will investigate the sensitivity analysis to test the stability and robustness of study findings based on the sample size of included trials, and study quality.

### Dissemination

We will publish this study on a peer-reviewed journal or a conference meeting.

## Discussion

This systematic review will allow us to separately synthesize the findings of RCTs and CCSs addressing the efficacy and safety of gabapentin for PLP after amputation in pediatric oncology. It will be based on the eligible published studies from the inception to the present and will allow us to assess study quality and analyze outcome data. It will also provide associated information on current knowledge of gabapentin for PLP after amputation in pediatric oncology. This will be conducted by accessing information without publication status and language limitations.

A variety of clinical trials suggested that gabapentin can relieve PLP after amputation in pediatric oncology. However, no systematic review is identified on investigating the efficacy and safety of gabapentin for PLP after amputation in children population comprehensively. Thus, this study represents the first systematic review to examine the efficacy and safety of gabapentin for PLP after amputation in children with oncology. We expect our results that should allow us to draw beneficial conclusions about the efficacy and safety of gabapentin for PLP after amputation in pediatric oncology, which may benefit both clinicians and future studies.

## Supplementary Information

**Additional file 1.** PRISMA-P Checklist.

## Data Availability

Data sharing is not applicable to this article as no datasets were generated or analyzed during the current protocol.

## Abbreviations

PLP: Phantom limb pain
GABA: Gamma-aminobutyric acid
PRISMA: Preferred Reporting Items for Systematic Reviews and Meta-Analysis
RCTs: Randomized controlled trials
CCSs: Case-controlled studies
CIs: Confidence intervals
MD: Mean difference
